# In Vivo Quantification of Creatine Kinase Kinetics in Mouse Brain Using ^31^P‐MRS at 7 T

**DOI:** 10.1002/nbm.70055

**Published:** 2025-05-01

**Authors:** Mohamed Tachrount, Sean Smart, Jason Lerch, Antoine Cherix

**Affiliations:** ^1^ Wellcome Centre for Integrative Neuroimaging, FMRIB, Nuffield Department of Clinical Neurosciences University of Oxford Oxford UK; ^2^ Neurosciences and Mental Health The Hospital for Sick Children Toronto Ontario Canada; ^3^ Department of Medical Biophysics University of Toronto Toronto Ontario Canada

**Keywords:** ^31^P‐magnetic resonance spectroscopy, 7 T, brain, creatin kinase, mouse, saturation transfer

## Abstract

^31^P‐MRS is a method of choice for studying neuroenergetics in vivo, but its application in the mouse brain has been limited, often restricted to ultrahigh field (> 7 T) MRI scanners. Establishing its feasibility on more readily available preclinical 7‐T scanners would create new opportunities to study metabolism and physiology in murine models of brain disorders. Here, we demonstrate that the apparent forward rate constant (*k*_f_) of creatine kinase (CK) can be accurately quantified using a progressive saturation‐transfer approach in the mouse brain at 7 T. We also find that a 20% reduction in respiration of anesthetized mice can lead to 36% increase in *k*_f_ attributable to a drop in cellular pH and mitochondrial ATP production. To achieve this, we used a test–retest analysis to assess the reliability and repeatability of ^31^P‐MRS acquisition, analysis, and experimental design protocols. We report that many ^31^P‐containing metabolites can be reliably measured using a localized 3D‐ISIS sequence, which showed highest SNR amplitude, SNR consistency, and minimal T_2_ relaxation signal loss. Our study identifies key physiological factors influencing mouse brain energy homeostasis in vivo and provides a methodological basis to guide future studies interested in implementing ^31^P‐MRS on preclinical 7‐T scanners.

## Graphical abstract

We demonstrate the feasibility of using ^31^P‐MRS to quantify creatine kinase activity in the mouse brain on preclinical 7‐T scanners. A 20% drop in respiration increased the apparent forward rate constant by 36%, likely due to altered pH and mitochondrial ATP production. Our study establishes reliable ^31^P‐MRS protocols and identifies key factors affecting brain energy homeostasis, providing a foundation for future research in murine models of brain disorders.

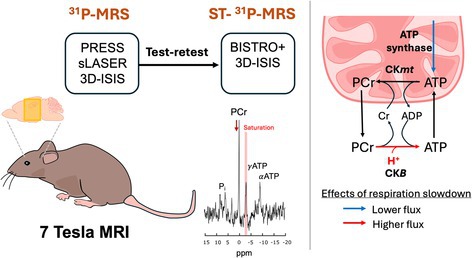

**Abbreviations**
^31^P‐MRS: phosphorous magnetic resonance spectroscopy
3D‐ISIS: image‐selected in vivo spectroscopy
ATP: adenosine triphosphate
BISTRO: B_1_‐insensitive train to obliterate signal
CK: creatine kinase
CMR_glc_
: cerebral metabolic rate of glucose
CMRO_2_
: cerebral metabolic rate of oxygen
CRLB: Cramér–Rao lower bound
CVs: coefficients of variation
FWHM: full width at half maximum
GPC: glycerophosphorylcholine
GPE: glycerophosphorylethanolamine
*k*_f_
: creatine kinase forward rate constant
NADH and NAD^+^
: nicotinamide adenine dinucleotide
OVS: outer volume suppression
PCho: phosphocholine
PCr: phosphocreatine
PDE: phosphodiesters
PE: phosphoethanolamine
P_i_
: inorganic phosphate
PME: phosphomonoesters
PRESS: point‐resolved spectroscopy
SD_BA_
: Bland–Altmann standard deviation
sLASER: semilocalized by adiabatic selective refocusing
SNR: signal‐to‐noise ratio
ST: saturation transfer
T_1_
: longitudinal relaxation time
T_2_
: transverse relaxation time
TE: echo time

## 1 Introduction

Phosphorous magnetic resonance spectroscopy (^31^P‐MRS) is a well‐established and time‐tested method for measuring energy metabolism in vivo. This technique allows for the quantification of phosphate‐containing molecules involved in energy homeostasis, such as phosphocreatine (PCr), adenosine triphosphate (ATP), inorganic phosphate (P_i_), and nicotinamide adenine dinucleotide (NADH and NAD^+^). Additionally, other phosphate‐containing molecules can be observed, including precursors and intermediates of phospholipid metabolism [1], such as phosphoethanolamine (PE) and phosphocholine (PCho), both often referred to as phosphomonoesters (PME), or glycerophosphorylethanolamine (GPE) and glycerophosphorylcholine (GPC), known as phosphodiesters (PDE). Moreover, intracellular pH can be indirectly measured from the chemical shift differences of P_i_ and PCr [2]. Finally, when combined with a saturation‐transfer (ST) preparation, ^31^P‐MRS provides a unique opportunity to measure the activity rates of key enzymes like creatine kinase (CK) and ATP synthase [3], both of which are critical in regulating cellular ATP production.

The foundations of ^31^P‐MRS were established over four decades ago, with the method tested and validated across various samples and organisms, including yeast [4], perfused rodent heart [5, 6], rodent brain [7, 8, 9] and, ultimately, human heart [10], and brain [11, 12]. Since then, ^31^P‐MRS has been refined and applied in numerous studies to investigate the metabolic basis of various disorders in humans [13, 14] and in animal models [15].

Despite the high potential of ^31^P‐MRS for studying in vivo metabolism, research on brain energetics in animal models has been scarce and often restricted to ultrahigh field (> 7 T) scanners. Acquiring ^31^P‐MRS spectra from small volumes like the rodent brain is technically challenging, making ultrahigh magnetic field scanners a valuable tool for enhancing signal‐to‐noise ratio (SNR) and spectral resolution. Although some studies using lower field scanners (< 7 T) have been attempted in mice, reporting metabolite changes in the brains of tumor‐bearing animals [16, 17, 18], in a scrapie model [19] or following overexpression of astroglial growth hormone [20], these studies have often been limited by their localization protocols and have primarily quantified only the metabolite peaks with the highest signals, such as PCr, ATP, or occasionally P_i_, with limited or no information on the reliability of the measured signals.

Ultrahigh field scanners (e.g., 9.4 or 14 T) have improved sensitivity and spectral resolution, enabling the study of neuroenergetic alterations in mouse models of neurodegenerative [21, 22, 23, 24], neurological [25, 26], or neuropsychiatric [27] disorders. This higher resolution has made it possible to acquire data on more complex biochemical processes, such as CK rate [22] measurements, or to distinguish between important metabolic pools, such as intracellular versus extracellular P_i_ or NADH versus NAD^+^ [27]. While the advantages of higher field scanners for ^31^P‐MRS are well established, including shorter longitudinal relaxation times (T_1_) and improved chemical shift separation [28], their prohibitive costs and technical challenges—such as B_0_ and RF inhomogeneities, especially with surface coils—have restricted their use to niche applications. Consequently, most recent method developments in mouse brain imaging have solely focused on using 9.4‐T scanners [29, 30, 31].

The feasibility and repeatability of ^31^P‐MRS measurements in the mouse brain using lower field strength preclinical scanners are less well understood. In this study, we explore the potential of using ^31^P‐MRS at 7 T to measure brain energy metabolism in the mouse brain, evaluating its benefits and reliability. We compare three standard sequences, assess the reliability and repeatability of the resulting metabolite quantifications, and examine the feasibility of using ST to quantify CK rates in vivo.

## 2 Materials and Methods

### 2.1 Animals

Male and female adult C57/BL6J mice obtained from in‐house breeding were housed in standard IVC cages in a normal 12‐h day‐light cycle environment at a temperature of 20°C–24°C and humidity of 46%–65%. Animals had ad libitum access to standard rodent chow diet and water. Animal care and experimental procedures were approved by the University of Oxford local ethical review committee and all experiments were carried out with approval by the UK Home Office, under the Animals (Scientific Procedures) Act 1986, and in compliance with the Animal Research: Reporting of In Vivo Experiments (ARRIVE) guidelines.

### 2.2 Experimental Design

#### 2.2.1 Test–Retest Analysis of ^31^P‐MRS

Six adult male C57BL/6J mice (35 ± 3 g, 33 weeks old) were scanned using ^31^P‐MRS on two occasions at 1‐week interval between scans. A test–retest analysis was done using three different sequences by comparing their respective SNR and the coefficients of variation (CVs) of their SNRs. The three sequences were applied in random order, but that order kept the same for each mouse on the two scan repetitions. Then, the performance of the best sequence was assessed by reporting the quantification error (Cramér–Rao lower bound [CRLB]) of each metabolite component for increasing acquisition times. This increase in acquisition time, from *t*_0_ to *t*_f_, was estimated by gradually increasing the number of included averages in random order from an initial scan of duration *t*_f_. The number of included averages is thus reported in time units for clarity throughout the manuscript. The CVs of the most reliable metabolite quantifications (PCr/ATP and P_i_/ATP) were then determined for between‐group, between‐session, and within‐session comparisons. The resulting CVs were ultimately used to perform power calculations for subsequent studies.

#### 2.2.2 Group Comparison for ST ^31^P‐MRS

Nine adult male (32 ± 2 g) and 11 female (23 ± 2 g) C57BL/6J mice (16 weeks old) were scanned using ST ^31^P‐MRS. The estimated CK forward rate constant (*k*_f_) was then correlated to physiological, biological, and acquisition‐related parameters. Animals were then grouped based on the biological parameter with strongest correlation coefficient, that is, respiration, using the arithmetic mean as a separation cutoff for the two groups.

### 2.3 In Vivo MRI Acquisition

Animals' anesthesia was induced with 2%–3% isoflurane in air mixture containing 30% O_2_. Animals were monitored during the entire scan for physiological parameters using a small animal monitoring system (SA Instruments Inc., New York, USA). Breathing rate per minute was maintained between 75 and 105 rpm by adjusting the isoflurane dose between 1% and 1.5%. Rectal temperature was kept at 36.8°C ± 0.5°C with a circulating heating water bath and assessed using a temperature rectal probe. Animals were scanned using a 7‐T (70/20) BioSpec MRI scanner (Bruker, Ettlingen, DE) using Paravision 360.1.1. A dual tuned ^31^P/^1^H surface coil with a single 10‐mm ^31^P loop (PulseTeq Ltd., Chobham, UK) was used as transceiver (Tx/Rx). A set of anatomical T_2_‐weighted images were acquired in axial, sagittal, and coronal orientations (TurboRARE, TE/TR = 11/2632.6 ms, RARE factor = 8) for localization and voxel placement for subsequent magnetic resonance spectroscopy (MRS) acquisitions.

### 2.4 In Vivo ^31^P‐MRS

For ^31^P‐MRS, a 160‐μL voxel (8 × 5 × 4 mm^3^) was placed in a brain area enveloping both hippocampus and hypothalamus. Shimming (MAPSHIM) was performed in the voxel to reach a water full width at half maximum (FWHM) of 26 ± 2 Hz. The acquisition was performed using point‐resolved spectroscopy (PRESS) [32], semilocalized by adiabatic selective refocusing (sLASER) [33], and image‐selected in vivo spectroscopy (3D‐ISIS) [34] in randomized order with similar acquisition parameters (Npoints = 1024, acquisition bandwidth [BW] = 40 ppm, TR = 4 s, averages/repetitions = 64 × 10, scan time = 42 min). All nonadiabatic RF pulses were based on Shinnar–Le Roux (SLR) type including excitation and refocusing pulses. The adiabatic pulses of ISIS and sLASER were of type hyperbolic secant (HS) with a bandwidth of 16 kHz and a pulse duration of 2 ms. When considering ATP and PCr chemical shift difference (i.e., 15 ppm or 1800 Hz at 120 MHz), the chemical shift displacement error (CSDE) was thus 11% for the refocusing pulses. The minimum allowed TE for each sequence was selected (TE_PRESS_ = 15 ms, TE_sLASER_ = 20 ms, TE_ISIS_ < 1 ms). The overall scanning time was 2h and 20 min per mouse. The RF power was optimized in phantoms and in vivo to adjust for maximal signal intensity. Optimal TR was assessed by measuring the T_1_ of PCr in phantoms using 3D‐ISIS with inversion recovery and by maximizing the SNR_PCr_ in one mouse in vivo. Focus was put on obtaining an optimal TR that benefits the signal resonances with highest intensity (ATP, PCr, and P_i_).

Spectra were processed (phase and B_0_‐drift corrections, and averaging) in jMRUI and analyzed with AMARES [35], using Lorentzian line‐shape modeling and constrained frequency, linewidth, and amplitude for each component (PCr, *γ*ATP, *α*ATP, *β*ATP, Pi_in, Pi_ex, PE, PCho, GPC, GPE, NAD_tot_, AND NAD^+^) with additional FID weighting in the first 20 points. The pH was determined as defined by AMARES, that is, Pi_in − PCr chemical shift difference. CRLB% were calculated by dividing the fit error (SD) obtained from AMARES quantification over the signal amplitude [36]. SNR of each scan was measured with MATLAB using the raw signal of PCr (peak height) and noise between 10 and 20 ppm. The SNR_PCr_ evolution with increasing acquisition number was fitted with a power equation (SNR = *a*·*N* ^*b*^) MATLAB (R2020a) using a Levenberg–Marquart algorithm.

### 2.5 ST ^31^P‐MRS

For ST ^31^P‐MRS, a 210‐μL voxel (7 × 5 × 6 mm^3^) was placed in the center of the brain. To limit the effect of contaminating signal from adjacent tissues, such as muscles, outer volume suppression (OVS) module using hyperbolic secant pulses was included (5‐mm‐thick slices with 1‐mm gap at each voxel side, 2‐ms HS pulse with 10‐kHz BW, CSDE of 18%), and shimming (MAPSHIM) was performed in the voxel to reach a water FWHM of 24 ± 2 Hz, followed by a ^31^P‐MRS acquisition using 3D‐ISIS (Npoints = 1024, acquisition BW = 40 ppm, TR = 8 s). The combination of OVS and ISIS was described before as the “OSIRIS” method [37]. Progressive ST experiment was performed using the B_1_‐insensitive train to obliterate signal (BISTRO) method [38], using a train of eight frequency selective 40‐ms HS2 pulses (BW = 100 Hz) at *γ*ATP offset, that is, −2.50 ppm [39] with variable RF field amplitude (with following scaling factors: 0.02, 0.04, 0.07, 0.14, 0.27, 0.49, 0.82, and 1). Saturation time was acquired in random order at five different values (336, 672, 1009, 2018, and 4710 ms), and a control spectrum was acquired with a mirrored saturation at +2.50 ppm. To provide a narrow and consistent saturation of *γ*ATP resonance, the effective saturation bandwidth of the BISTRO protocol was compared to similar protocols using constant flip angles using HS2 pulses or sinc3 pulses in phantoms. Each ST experiments lasted ~2 h per subject. After spectra postprocessing in jMRUI, the PCr signals were fitted using a single Lorentzian line shape in AMARES. A monoexponential function was fitted (MATLAB) to the relative PCr signal (*M*_PCr_) decay as a function of saturation time (*t*_sat_), to determine both *k*_f_, the pseudo–first‐order forward reaction PCr → ATP rate constant (*k*_PCr→ATP_), and T_1_, the apparent T_1_ of PCr [22], using following equation:

(1) $$\frac{M_{\mathrm{PCr}}\left(t_{\mathrm{sat}}\right)}{M_{\mathrm{PCr}}\left(t_{0}\right)}=\left(1-k_{f}\cdot T_{1}\right)+k_{f}\cdot T_{1}\cdot \exp^{-\frac{t_{\mathrm{sat}}}{T_{1}}}.$$

The pH was determined from the chemical shift difference between P_i_ and PCr on the averaged spectrum for each mouse.

### 2.6 Statistics

Statistics were all performed with GraphPad Prism 9 (GraphPad Software, San Diego, CA, USA). All values are given as mean ± SD unless stated otherwise. *p* < 0.05 were considered statistically significant. Sequence SNRs were compared using a Kruskal–Wallis test with Dunn's multiple comparison test. For all three sequences, the between‐session CV (i.e., SD/mean) was assessed for increasing scanning time, that is, for increasing number of spectra averages, using a Bland–Altmann standard deviation (SD_BA_) [40].

(2) $${\mathrm{SD}}_{\mathrm{BA}}=\sqrt{\frac{\sum {\left(x_{1}-x_{2}\right)}^{2}}{2n}},$$

where *x*_1_ and *x*_2_ represent the two repeated measurements of the two sessions for the *n* subjects.

For 3D‐ISIS, the metabolite's CVs were computed for between group, within session, and between session with increasing scanning time. Between‐group SD was calculated from Session 1, within‐session SD was calculated from the average quantification for all mice (Session 1 + 2) over the time of acquisition for three different time resolutions (4, 8, and 13 min), and finally, the between‐session CV was determined using a Bland–Altmann SD (SD_BA_).

For the power calculations, the following equation was used [41]:

(3) $$N=\frac{{\left(Z_{1-\alpha /2}+Z_{\beta -1}\right)}^{2}\cdot {\mathrm{CV}}^{2}}{{\left(\%\Delta \right)}^{2}{\left(1-\frac{3}{4n-9}\right)}^{2}}.$$

With *N*, the number of subjects per group required, *Z* scores for *α* = 0.05 and *β* = 20% ($Z_{1-\alpha /2}=1.96$ and $Z_{\beta -1}=0.8416$), CV the coefficient of variation, and %Δ the relative change expected. Hedge's *g* was used to correct for small sample size bias inherent to Cohen's *d*. Hedge's *g* is given as [42]

(4) $$g=d\cdot \left(1-\frac{3}{4n-9}\right).$$

With Cohen's $d=\frac{\%\Delta }{\mathrm{CV}}$ and *n* is the total number of samples in the effect size estimation.

Fitting of the creatine CK rate constant *k*_f_ was done using MATLAB (R2020a) using a Levenberg–Marquart algorithm. Correlations between *k*_f_ and other parameters were estimated using Pearson correlation coefficients. Group comparisons between higher and lower respiration rates were done using Student's *t* tests.

## 3 Results

### 3.1 Localized ^31^P‐MRS in Mouse Brain at 7 T Using 3D‐ISIS Leads to Higher SNR and Better Repeatability Than PRESS and sLASER

**FIGURE 1:**
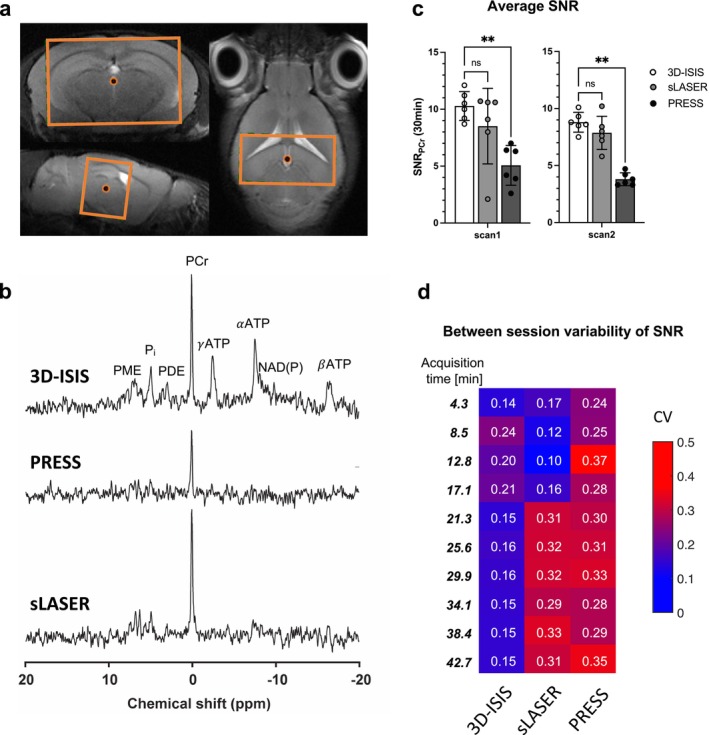
Sequence comparison for localized ^31^P‐MRS in mouse brain at 7 T. (a) Anatomical T_2_‐weighed MRI with voxel placement (orange) in the mouse brain (8 × 5 × 4 mm^3^). (b) Example of ^31^P‐MRS spectra acquired using 3D‐ISIS, PRESS, and sLASER in the same mouse. Spectra were acquired with 10 repetitions of 64 average (8 averages of 8 acquisitions per cycle for ISIS), all led to 43‐min acquisition time and are shown with 10‐Hz apodization. (γ,α, and β)ATP: gamma‐, alpha‐, and beta‐adenosine triphosphate; NAD(P): nicotinamide adenine dinucleotide (phosphate); PCr: phosphocreatine; PDE: phosphodiesters; P_i_: inorganic phosphate; PME: phosphomonoesters. (c) Average SNR (PCr peak) for each scan session. Kruskal–Wallis test with Dunn's multiple comparison test **p < 0.005. (d) Coefficients of variation (CVs) of the SNR (PCr) of each sequence at increasing number of averages or acquisition time.

For each mouse, a set of spectra was acquired using each of the three ^31^P‐MRS sequences, that is, 3D‐ISIS, PRESS, and sLASER to assess their performance in mouse brain at 7 T. Each sequence led to a measurable PCr signal (Figure 1a,b), with clear visual differences at the level of metabolites with short T_2_‐relaxation (e.g., γATP, αATP, and βATP). The SNR of the PCr peak was measured in each spectrum (Figure 1c), indicating comparable values between 3D‐ISIS and sLASER (ns) but a better performance of 3D‐ISIS over PRESS (*p* < 0.005) in both scan sessions. No difference was observed in the relationship of SNR_PCr_ with time of acquisition, with a near‐square root relationship for each sequence (*b* ≈ 0.4; Figure S1). Finally, to test the repeatability of each sequence, the CV of the SNR was calculated when increasing the number of included averages of each scan (Figure 1d). This analysis indicated that 3D‐ISIS has the lowest CV of SNR_PCr_, which stabilized at 15%–16% for acquisitions > 20 min of scan. Interestingly, while sLASER performed better for short acquisitions (< 20 min) with a CV under 20%, its repeatability deteriorated for longer scans.

### 3.2 Acquisition With 3D‐ISIS Leads to Reliable and Repeatable Quantification of Most ^31^P‐Metabolites at 7 T

**FIGURE 2:**
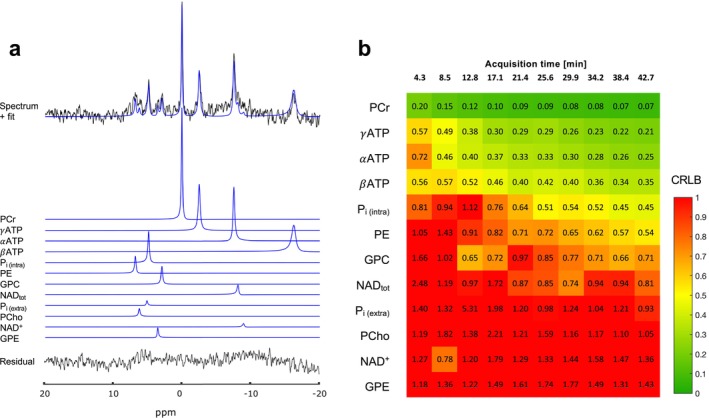
Quantification and measurement errors of ^31^P‐MRS using 3D‐ISIS. (a) Typical 3D‐ISIS spectrum (black) from 160‐μL voxel and fit (blue) from AMARES (jMRUI). (b) Average quantification error (CRLB) resulting from AMARES fit for each individual component for increasing acquisition time (number of spectra averages). (γ,α, and β)ATP: gamma‐, alpha‐, and beta‐adenosine triphosphate; GPC: glycerophosphorylcholine; GPE: glycerophosphorylethanolamine; NAD^+^: nicotinamide adenine dinucleotide; NAD_tot_: nicotinamide adenine dinucleotide (1/2NAD(P)^+^ + NAD(P)H); PCho: phosphocholine; PCr: phosphocreatine; PE: phosphoethanolamine; P_i(extra)_: extracellular inorganic phosphate; P_i(intra)_: intracellular inorganic phosphate.

We then looked at how well ^31^P‐containing metabolites could be quantified using the 3D‐ISIS sequence (Figure 2a,b). The quantification error of high‐signal resonances, such as PCr, P_i_, and ATP resonances (γATP, αATP, and βATP), dropped and stabilized after the first 20 min of scan, leading to relatively low CRLB: CRLB(PCr) = 7%–9%, CRLB(γATP) = 21%–30%, CRLB(αATP) = 25%–33%, CRLB(βATP) = 34%–42%, and CRLB(P_i_) = 45%–54%. Within 45 min of acquisition, the CRLB of PE, GPC, and total nicotinamide adenine dinucleotide (NAD_tot_) also dropped and stabilized: CRLB(PE) = 54%–65%, CRLB(GPC) = 66%–77%, and CRLB(NAD_tot_) = 74%–94%. Finally, the CRLBs of GPE, NAD^+^, and phosphocholine (PCho) were all above 100% within 45 min of acquisition in the 160‐μL voxel indicating poor quantification performance. Importantly, the data quality did not allow to distinguish the intracellular (P_i(intra)_) component (at 4.9 ppm) from the extracellular (P_i(extra)_) component (at 5.3 ppm) of P_i_ when fitting them separately.

**FIGURE 3:**
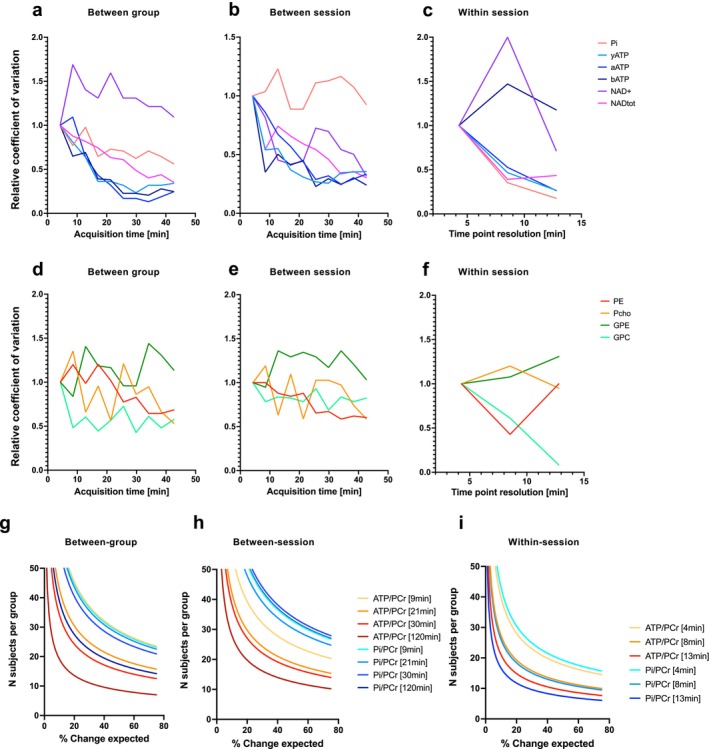
Test–retest analysis of individual ^31^P‐MRS metabolites for repeatability assessment and power calculations. (a–f) Relative coefficient of variation (CV) for each metabolite as a function of acquisition time (number of spectra averages), when considering a two‐group comparison (a,d), two‐session repetition (b,e), and within‐session fluctuation (c,f). (g–i) Associated statistical power calculations for ATP/PCr and P_i_/PCr ratios at different acquisition times. The CV at 120 min was estimated from extrapolating the CV decay in Figure 3a,b.

High CRLB values are not necessarily suggesting poor quantification, as low‐concentration metabolites will inevitably have higher CLRBs than high‐concentration ones [43]. To take this effect into account, we also looked at the change in relative CV of the measured concentration (relative to PCr) for each metabolite with increasing number of averages (Figure 3a–f) as a measure of quantification reliability. Absolute CV comparison for 40‐min acquisitions were below 50% for most metabolites, except GPE and GPC, for “between‐group” and “between‐session” experiments (Table S1) and were below 20% for a “within‐session” design, except for GPE. Results for “between‐group” comparison indicated that the CV of P_i_/PCr and (γ, α, and β)ATP/PCr dropped by 60% within 20 min of acquisition, while it dropped by ~50% for NAD^+^_tot_ and P_i_ within 40 min. Among all other metabolites, only the CV of GPC/PCr and PE/PCr dropped by ~50% within 40 min of acquisition (Figure 3d). Between‐session variation was comparable for ATP/PCr but worse for P_i_/PCr, which did not show any improvement with increasing acquisition time (Figure 3b). When looking at within‐session comparison, increasing the acquisition time resolution improved the CVs of most metabolites except, βATP, GPE, and PCho (Figure 3c,f). Power calculations were then performed using these CV, on the highest signals, that is, γATP/PCr and P_i_/PCr, to estimate the potential sample size of animals required for three experimental designs (i.e., between group, between session, and within session) (Figure 3g–i). Power calculations for a within‐session comparison suggest that changes of ATP/PCr ratio above 25% could be easily detected for a 13‐min timepoint resolution using around 12 animals. This confirms that reliable measurement of changes in PCr signal during a ST experiment with a spectral average acquisition of 13 min for each saturation time can be achieved.

### 3.3 ST ^31^P‐MRS Is Feasible for Measuring the Forward CK Rate Constant in Mouse Brain at 7 T

**FIGURE 4:**
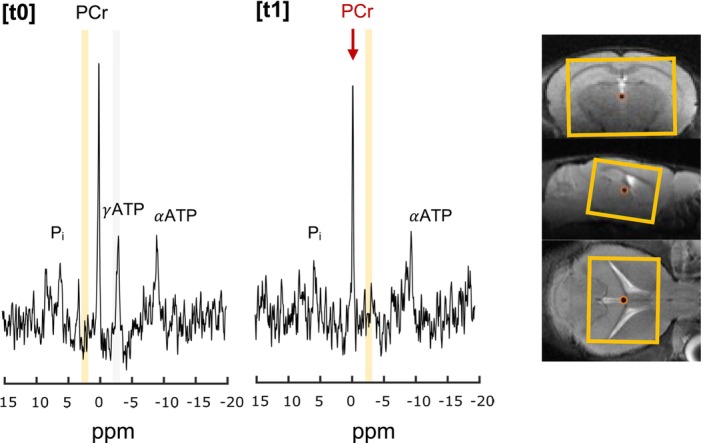
BISTRO ^31^P‐MRS saturation transfer in mouse brain at 7 T. Typical ^31^P‐MRS spectrum before (Sat_offset_(*t*_0_) = 2.50 ppm) and after (Sat_offset_(*t*_1_) = −2.50 ppm) saturation of γATP in mouse brain (orange; 7 × 5 × 6 mm^3^). Spectra shown with 10‐Hz Lorentzian apodization.

We then explored the feasibility of measuring CK activity in mouse brain using a progressive ST experiment. This was performed in a bigger voxel (210 μL) to maximize PCr and ATP signals. BISTRO pulse train led to a clean saturation with steady saturation bandwidth as measured in phantom, compared to the two other protocols, even at long saturation times (Figure S2). Notably, the effective saturation bandwidth was generally broader than the expected 100 Hz, likely due to the cumulative effects of RF pulses, which intensified with longer saturation time (saturation train). Overall, BISTRO resulted in an efficient saturation of γATP resonance and drop of PCr with minimal or no bleed over in the in vivo experiments (Figure 4).

### 3.4 ST ^31^P‐MRS Detects Physiologically Relevant Changes in Brain CK Function in Mouse Brain

**FIGURE 5:**
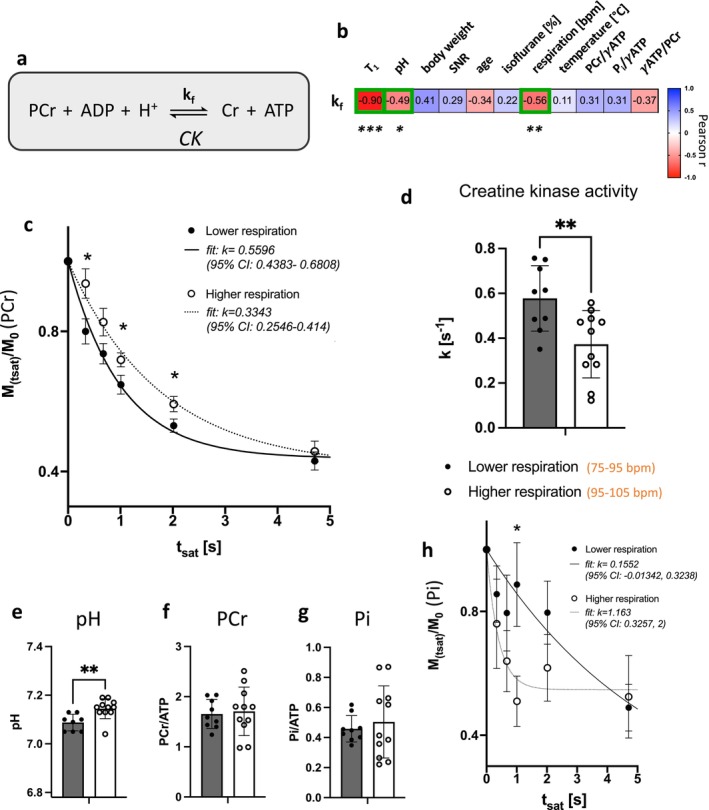
Breathing rate‐dependent changes in CK activity can be measured in mouse brain using saturation‐transfer experiments at 7 T. (a) Chemical reaction catalyzed by creatine kinase (CK). *k*_f_: CK pseudo–first‐order forward rate constant. (b) Correlations of *k*_f_ with physiological, biological, and acquisition‐related parameters (Pearson correlation coefficient) ***p < 0.0001, **p = 0.013, *p = 0.047. (c) Group average of the relative PCr signal (*M*_(tsat)_/*M*_0_) with increasing ATP saturation times (*t*_sat_) for higher versus lower respiration rate, following progressive saturation‐transfer experiment. Data is shown with exponential fit for *k*_f_ (group average) with 95% confidence interval (CI). Student *t* test for individual time points, *p < 0.05. (d–g) Group comparisons of *k*_f_ (d), pH (e), phosphocreatine (f) and inorganic phosphate (g) for mice with higher versus lower respiration rate. **p < 0.01. (h) Group average of the relative P_i_ signal (*M*_(tsat)_/M_0_) with increasing ATP saturation times (*t*_sat_).

Finally, to test the biological relevance and applicability of ST ^31^P‐MRS acquisitions in the mouse brain, we investigated the relationship between the CK forward rate constant (*k*_f_) with various physiological, biological, and acquisition‐related parameters. The pseudo–first‐order CK forward rate constant (*k*_f_; Figure 5a) was determined for each mouse by fitting an exponential decay on the PCr‐saturation time course. The resulting *k*_f_ were then correlated with the various parameters and revealed a strong correlation with T_1_ (*R* = −0.90, *p* < 0.0001). This indicates a strong dependence of these two variables, suggesting difficulty to infer them independently from the fitting approach (Figure 5b). Nevertheless, *k*_f_ was also correlated with the respiration rate (*R* = −0.56, *p* = 0.01) and the pH (*R* = −0.49, *p* = 0.047) indicating a physiologically relevant relationship. Alternatively, no significant correlations were found for body weight, age, isoflurane dose, temperature, and SNR. Importantly, *k*_f_ did not significantly correlate with the metabolite ratios γATP/PCr and P_i_/PCr, supporting the idea that the metabolite concentrations remain in steady‐state independently of the CK flux. No group differences in *k*_f_ were also observed between male and females (ns).

Respiration rate, determined by the anesthetic depth, could potentially influence changes in CK activity through a local effect on oxygenation and pH. We thus further tested whether differences in *k*_f_ values could be explained by respiration throughout the ^31^P‐MRS acquisition (Figure S3) by dividing the dataset into two groups, classified as “higher” or “lower” average breathing rates relative to the arithmetic mean of the whole dataset. When fitting the exponential decay model to the group average PCr‐saturation data, we observed a lower *k*_f_ for the higher respiration group (*k* = 0.33 [s^−1^], 95% CI: 0.25–0.41 [s^−1^]) compared to the lower respiration group (*k* = 0.60 [s^−1^], 95% CI: 0.44–0.68 [s^−1^]). When fitting the exponential decay model to the PCr‐saturation data of each mouse individually, we found that animals with lower respiration rate (75–95 bpm) had significantly higher *k*_f_ values (0.58 ± 0.15 [s^−1^] vs. 0.37 ± 0.15 [s^−1^], 36% increase, *p* = 0.007) compared to those with higher respiration rate (95–105 bpm). This group comparison also revealed smaller pH values in the lower respiration group (*p* = 0.005), while no difference was observed in PCr/ATP, P_i_/ATP and T_1,sat_ (Figures 5f,g and S4). CK forward rate constant is sensitive to the local concentration of protons (H^+^); thus, a drop in pH is expected to lead to an increase in the measured *k*_f_ (Figure 5a). We also investigated the potential effects on the ATP‐synthase rate by looking at the relative decay of P_i_ signal following the same ATP saturation scheme (Figure 5h). When fitting an exponential decay to the average *M*_sat_/*M*_0_ time courses of each group, we observed a lower rate of ATP synthase in mice with lower respiration (*k*_Pi⟶ATP_ = 0.15; 95% CI: −0.01 to 0.32) compared to higher respiration (k_Pi⟶ATP_ = 1.16; 95% CI: 0.33–2.00). Despite the lack of precision on estimating those two parameters, the two distinct 95% confidence intervals suggest a group difference. These findings demonstrate how the CK reaction is modulated in the mouse brain by local pH shifts, which may arise from physiological and metabolic changes induced by anesthesia.

## 4 Discussion

This study aimed to investigate the feasibility and repeatability of ^31^P‐MRS to study mouse brain metabolism through ST experiments on a 7‐T scanner. We demonstrate that, under the proposed protocol, the quantification of most ^31^P‐metabolites is consistent and robust, enabling a reliable assessment of CK activity in the mouse brain. Using this methodology, we identify key neuroenergetic fluctuations that occur in conjunction with physiological changes in the animal under isoflurane anesthesia in vivo.

Detecting of ATP and P_i_ resonances in ^31^P‐MRS can be challenging due to their relatively short transverse relaxation times (T_2_) [44]. Although accurately measuring T_2_ can be difficult, studies have observed a trend toward reduced T_2_ values with increasing scanner field strength [45, 46], suggesting that 7‐T scanner might allow for longer echo times (TE) compared to higher field scanners (> 7 T). To explore this, we compared three sequences that differ in their acquisition schemes, including TE. ISIS, which is based on a multiacquisition of free induction decay, allows for the shortest delay between spin excitation and signal acquisition [34], making it sensitive to fast T_2_‐relaxing metabolites. However, its reliance on spectral subtraction for localization makes it inherently susceptible to motion artifacts [47]. On the other hand, conventional single‐shot acquisition methods such as PRESS or sLASER result in longer TE (15–20 ms in our case) but may offer more stable signal acquisition, potentially better suited for long scans. sLASER, in particular, provides improved spatial localization and reduces chemical shift displacement artifacts [33]. While inhomogeneous radiofrequency (B_1_) fields can be problematic with surface coils, the use of adiabatic pulses in sLASER achieves more uniform and efficient signal refocusing, leading to narrower spectral lines and improved resolution. Despite producing comparable PCr signal, our results indicate that sLASER still struggles to detect short T_2_ metabolites at 7 T. Notably, the CV of the PCr signal between sessions was higher with sLASER than with 3D‐ISIS, particularly over longer periods, suggesting reduced signal stability. This result is somewhat unexpected, as ISIS is generally considered more sensitive to motion compared to other localization techniques. One possible explanation lies in the reliance of sLASER on transverse magnetization refocusing using pairs of adiabatic RF pulses. The nonlinear phase induced by the first RF pulse is refocused by the second one. Motion could introduce a phase that cannot be refocused by the second pulse. In contrast, ISIS avoids these issues by not relying on transverse refocusing and is thus less reliant on crusher gradients for signal dephasing, which may influence the SNR and thus the repeatability. Although the difference in SNR between ISIS and sLASER was not statistically significant, it may have contributed to the observed disparity in repeatability. We also considered the potential impact of RF coil heating or power amplifier instability, but no clear signs of these effects were observed in the spectra.

The 3D ISIS sequence provided a reliable quantification of key energy‐related ^31^P‐metabolites in the mouse brain at 7 T. Metabolites with clearly identifiable peaks, such as ATP, PCr, and P_i_ achieved satisfactory quantification after 20 min of acquisition. Our results also suggest that some low‐concentration metabolites with high CRLBs, such as NAD_tot_, PE, and GPC can be measured convincingly within an hour. CRLB estimate the reliability of the quantification [48], but interpreting them as a percentage of the estimated concentration can be misleading, as higher CRLB values do not necessarily indicate poor fitting [43]. Instead of focusing solely on absolute CRLB values, we also considered whether CRLBs decreased with improved SNR when acquisition time, or the number of spectral averages, is increased. We further assessed whether higher number of averages improved the repeatability (relative CV) of the metabolite quantifications. However, some metabolites, such as PCho and GPE, remained unreliable with consistently high CRLBs and CVs. Additionally, we were unable to distinguish between NAD^+^ and NADH or between intracellular and extracellular P_i_ pools. Increasing B_0_ field strength reduces the spin–lattice (T_1_) relaxation time of ^31^P resonances [49] and their peak linewidths [28] in ^31^P‐MRS acquisitions. Higher field scanners (> 7 T) may thus be necessary to assess these low‐concentration and closely adjacent resonances.

Our power calculations suggest that typical preclinical group sizes (10–12 mice) may suffice to detect metabolite concentration changes of around 20% with standard commercially available equipment. A between‐session design exhibited the highest variability between metabolic readings, likely due to physiological factors such as anesthesia‐induced metabolic changes or circadian and physiological variations. However, a “within‐session” design revealed minimal variability, indicating that this method effectively detects changes occurring during the scan. This protocol is therefore well suited for ST experiments.

Using a BISTRO saturation sequence, we were able to measure CK forward rate constant (*k*_PCr→ATP_ or *k*_f_) in the mouse brain. BISTRO, which uses a gradual increase in the saturation RF pulse power, creates a sharp saturation profile that reduces bleedover on adjacent resonances, especially critical at long saturation times [50]. Fitting an exponential function to the relative PCr decay following saturation of *γ*ATP allowed us to infer a single *k*_f_ value for each mouse. However, assessing ATPase activity and fitting the model to the relative P_i_ signal was only possible by averaging across subjects, making group comparisons challenging. The resulting *k*_f_ values were all in the physiological range, (*k*_f_ = 0.46 ± 0.18 s^−1^) and distinguished mice based on their respiration regimes. A previous study reported *k*_f_ values in the brain of wild‐type mice at 36 (*k*_f_ = 0.45 ± 0.08 s^−1^) and 72 weeks (*k*_f_ = 0.49 ± 0.04 s^−1^) [22] of age. The authors found no significant group difference in *k*_f_ between wild‐type and 5xFAD mouse models of Alzheimer's disease, despite an overall inverted trend in *k*_f_ values (0.49 ± 0.04 s^−1^ at 36 weeks and 0.43 ± 0.06 s^−1^ at 72 weeks). While the ages and average *k*_f_ values of our cohort align with that study, the higher variability observed in our data may be due to a broader range of animal respiration rates. However, the previous study did not report individual respiration regimes, making this difficult to confirm. Finally, we observed high correlation between the two fitted parameters, T_1_ and *k*_f_, suggesting that uncertainty in their estimation may contribute to the variability observed.

We found that relatively small changes in respiration rate (10%–20%) in anesthetized mice can lead to significant changes in brain energy metabolism, detectable by ^31^P‐MRS at 7 T. A lower respiration rate was associated with a 36% increase in CK *k*_f_ rate and an opposite trend for the ATPase activity. The CK‐catalyzed reaction (Figure 5a) helps maintain steady intracellular ATP levels when oxygen levels drop or mitochondrial ATP production decreases. The build‐up of [H^+^], seen as a drop in pH due to increased glycolysis, pushes the reaction toward ADP phosphorylation via PCr through CK activity. This mechanism maintains ATP levels constant when oxidative phosphorylation is compromised. Reduced intracellular O_2_ levels or build‐up of HCO_3_^−^ may drive this metabolic switch resulting from lower animal breathing rate. Importantly, while the ATPase rates (k_Pi→ATP_) inferred from this analysis provided valuable insights into group differences, their absolute values should be interpreted with caution due to the inherent variability in the estimates.

Various physiological changes, such as hypoxia, hypoglycemia, or temperature variations, have been reported to influence CK rate [51, 52]. Brain maturation has also been associated with increase in CK activity, particularly the mitochondrial isoenzyme [53, 54]. Regional differences in CK activity have been observed, with higher rates in gray matter compared to white matter [55]. CK fluxes are also affected by age, psychiatric disorders and neurodegenerative diseases [56, 57]. Studies using ^31^P‐MRS on rat brains have highlighted the influence of anesthetic regime and dosage on the overall brain energy production. For example, Bresnen and Duong reported a decrease in CK rate constant with higher isoflurane doses (1.2% vs. 2%) [58]. Sauter and Rudin found that *k*_f_ correlates with brain EEG activity, with values ranging from 0.10 to 0.70 [s^−1^] when modulating brain activity with bicuculline or thiopental under halothane anesthesia [59]. Du et al. observed that deeper anesthesia in rats leads to lower ATPase and CK activities due to reduced CMR_glc_ and CMRO_2_ [60]. Importantly, they did not observe a drop in brain tissue pH due to the delivered anesthetic dose. While these studies did not examine the effects of respiration rate, they also focused on the rat, which require less frequent lung ventilation than mice. Consequently, ^31^P‐MRS measurements in rats may be less sensitive to fluctuations in breathing rate than in mice. While a switch from oxidative respiration to glycolysis in mouse brains may not significantly change ATP or other metabolites that maintain homeostasis, sustaining a constant breathing rate in anesthetized animals may be crucial to minimizing variability in brain energetic organization. Our results underscore the challenges of extrapolating findings from rat studies to mouse models due to significant physiological and metabolic differences between the two species.

## 5 Future Perspectives and Limitations

Mouse models are essential tools in biomedical research, contributing significantly to our understanding of brain function and dysfunction in human diseases. This study provides lays the groundwork for future developments aimed at improving the applicability and interpretation of ^31^P‐MRS in small rodents. While our work aligns well with a previous report showing that ISIS outperforms other conventional single‐voxel MRS sequences for ^31^P‐MRS at 9.4 T in the human brain [46], we did not investigate all the sequences included in that comparison—such as stimulated echo acquisition mode (STEAM)—which demonstrated comparable SNR per unit time to ISIS for metabolites with very short T_2_ relaxation times. Further sequence optimizations should be explored, such as implementing adiabatic excitation pulses or use of other ST protocols which may help better decouple T_1_ from *k*_f_ (e.g., FAST [61] or 3D‐ISIS with inversion recovery). We used adiabatic HS pulses for reliable inversion in ISIS at 7 T to ensure uniform saturation despite B_0_ and B_1_ inhomogeneities. While effective, this approach requires high RF power, and more energy‐efficient pulse designs could be explored for future studies to improve signal stability. Refining the modeling approaches may help improve the precision of flux estimates, particularly for the k_Pi⟶ATP_ parameter, which exhibited notably low precision. Future work could focus on incorporating non‐Gaussian noise, arising from the spectral decomposition, into the fitting process to further improve parameter estimation. Future studies characterizing the different parameters that influence CK activity in the mouse brain, such as using different anesthetics or controlling blood gases and glycemia, will help us better understand the neuroenergetic processes underlying mouse brain function.

## 6 Conclusion

This study confirms that 7‐T MRI scanners can achieve reliable measurements using the proposed ^31^P‐MRS protocol for both metabolite peaks and CK kinetics quantifications in the mouse brain. The observed relationship between respiration and brain *k*_f_ highlights the importance of careful monitoring the animal's physiology under isoflurane anesthesia.

## Author Contributions

A.C. designed the study. M.T., A.C., and S.S. optimized the protocol. A.C. acquired and analyzed the data. A.C. and M.T. interpreted the data. A.C. drafted the manuscript. M.T., A.C., S.S., and J.L. assisted in revising the manuscript and approved the final version.

## Ethics Statement

The authors declare no conflicts of interest with respect to the research, authorship, and/or publication of this article. This research was funded in part, by the Wellcome Trust (203139/Z/16/Z and 203139/A/16/Z). For the purpose of open access, the author has applied a CC BY public copyright license to any Author Accepted Manuscript version arising from this submission.

## Supporting information


**Table S1.** Absolute coefficient of variation in the test–retest analysis of individual ^31^P‐MRS metabolites. Calculated coefficients of variation (CVs) for each metabolite after 43 min of acquisition for “between‐group” and “between‐session” experiments and for 13‐min time points for “within‐session” experiments.
**Figure S1.** Relationship of SNR_PCr_ with increasing number of acquisitions. (a) Relationship between the SNR of phosphocreatine (SNR_PCr_) and number of acquisition averages (represented as acquisition time here). (b) Fitted parameters of a power equation (SNR = *a*·*N* ^*b*^, where *N* is the number of acquisition) for individual mice. No sequence effect was observed for parameter *b* (*F*(2, 20) = 1.89, *p* = 0.18), but only for parameter a (*F*(2, 20) = 4.47, *p* = 0.02), RM one‐way ANOVA, *p < 0.05, Bonferroni’s post hoc test.
**Figure S2.** Effect of long saturation time on saturation pulse bandwidth. Saturation pulse bandwidth comparison in phantom using BISTRO (green), HS2 pulse with fixed amplitude (orange) or sinc3 pulse with fixed amplitude (blue) at various saturation times.
**Figure S3.** Stability of respiration during ST ^31^P‐MRS acquisition. (a) Breathing of higher and lower respiration groups. (b) Individual time courses of breathing rates for mice in the higher and lower respiration groups. (c) comparison between initial (first 20 min) and final (final 20 min) breathing rates for individual mice in each respiration groups. Group effect (*F*(1, 16) = 14, *p* = 0.002), time effect (*F*(1, 16) = 6.58, *p* = 0.02), repeated‐measure two‐way ANOVA, nonsignificant (ns) Bonferroni’s post hoc test.
**Figure S4.** Apparent relaxation time T_1_ for the higher and lower respiration groups (ns, not significant).

## Data Availability

The data that support the findings of this study are available from the corresponding author upon reasonable request.
