# Supplementary material for: In Vivo Quantification of Creatine Kinase Kinetics in Mouse Brain Using 31P‐MRS at 7 T
Source: NMR Biomed. 2025 May 1;38(6):e70055. doi: 10.1002/nbm.70055 (PMC12046211; doi:10.1002/nbm.70055)

Supplementary Material

Supplementary Table 1

	**Coefficients of variation (CV %)**		
	Between group	*Between session*	*Within session*
**γATP**	0.16	0.19	0.04
**αATP**	0.13	0.18	0.05
**βATP**	0.24	0.24	0.20
**Pi**	0.27	0.44	0.03
**NAD^+**	0.46	0.28	0.10
**NAD_{tot}**	0.29	0.29	0.10
**PE**	0.35	0.39	0.14
**PCho**	0.43	0.37	0.19
**GPE**	0.60	0.71	0.34
**GPC**	0.61	0.61	0.03
**pH**	0.01	0.007	0.0034
**Mg^{2+}**	0.23	0.36	0.08

Supplementary Figure 1

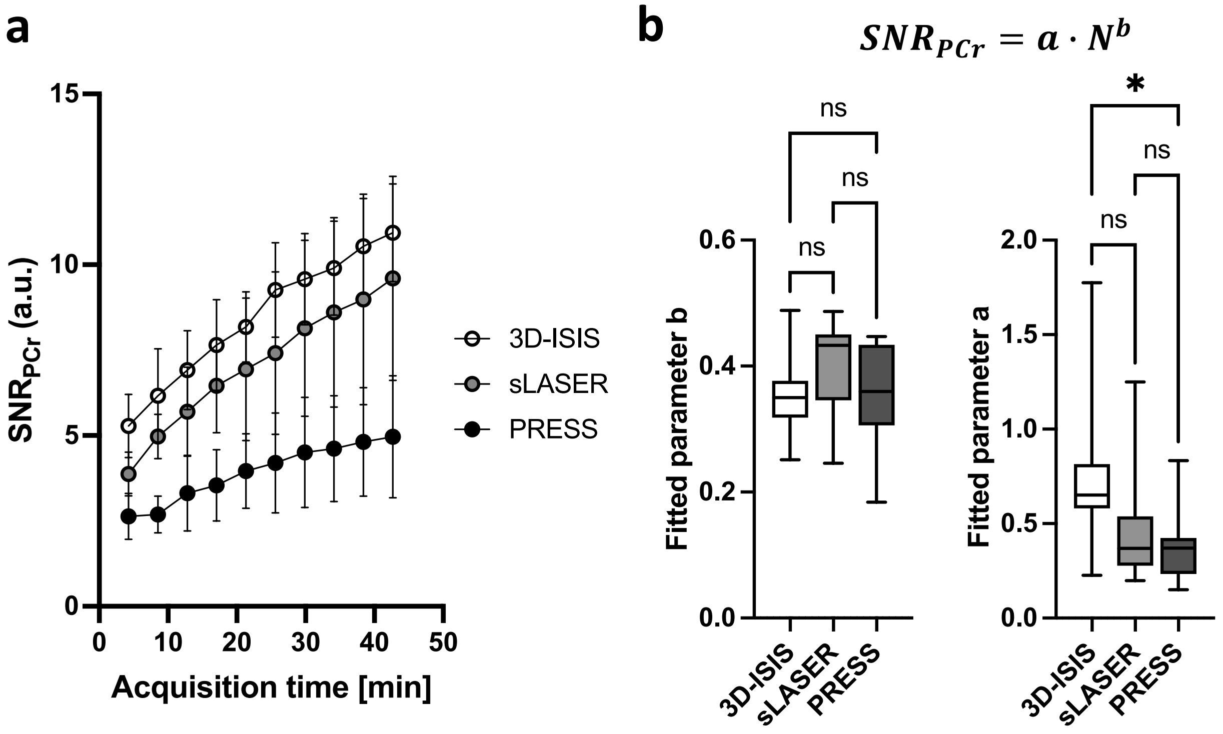

Supplementary Figure 2

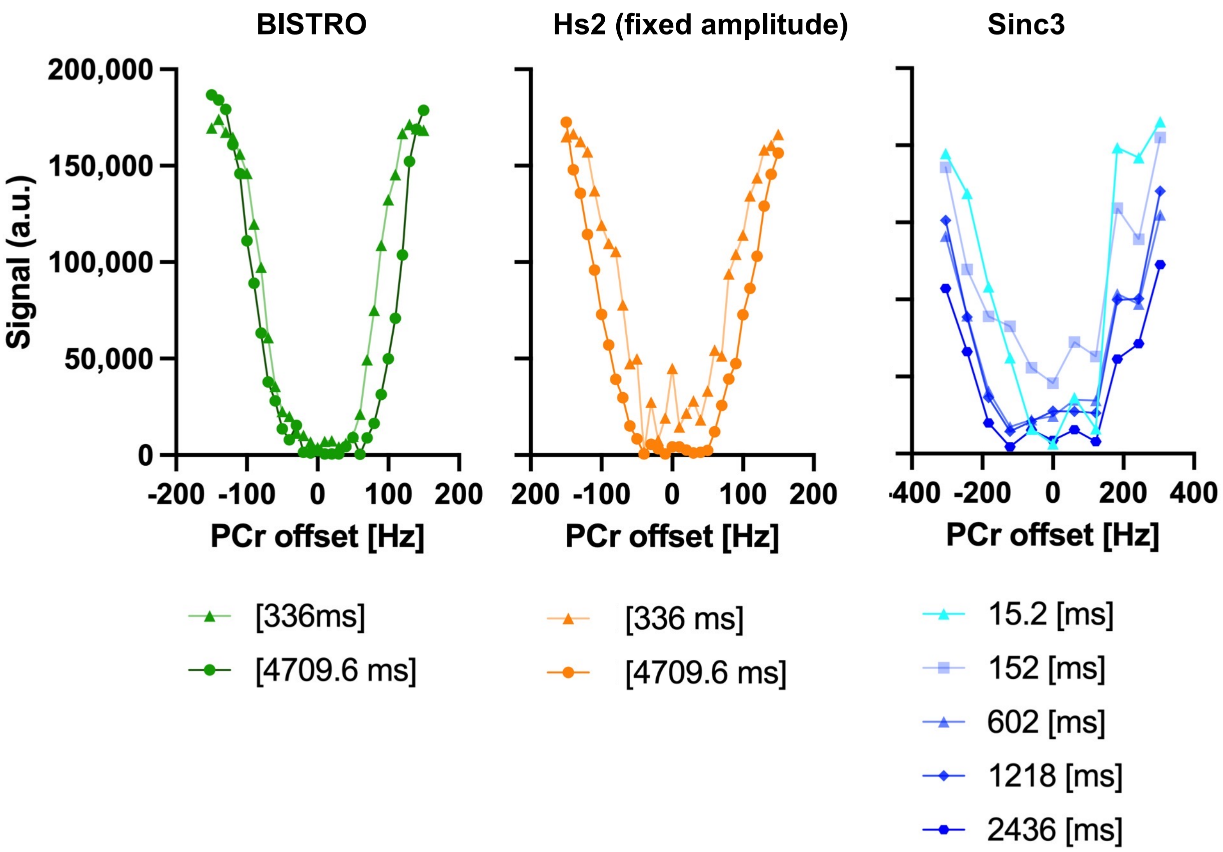

Supplementary Figure 3

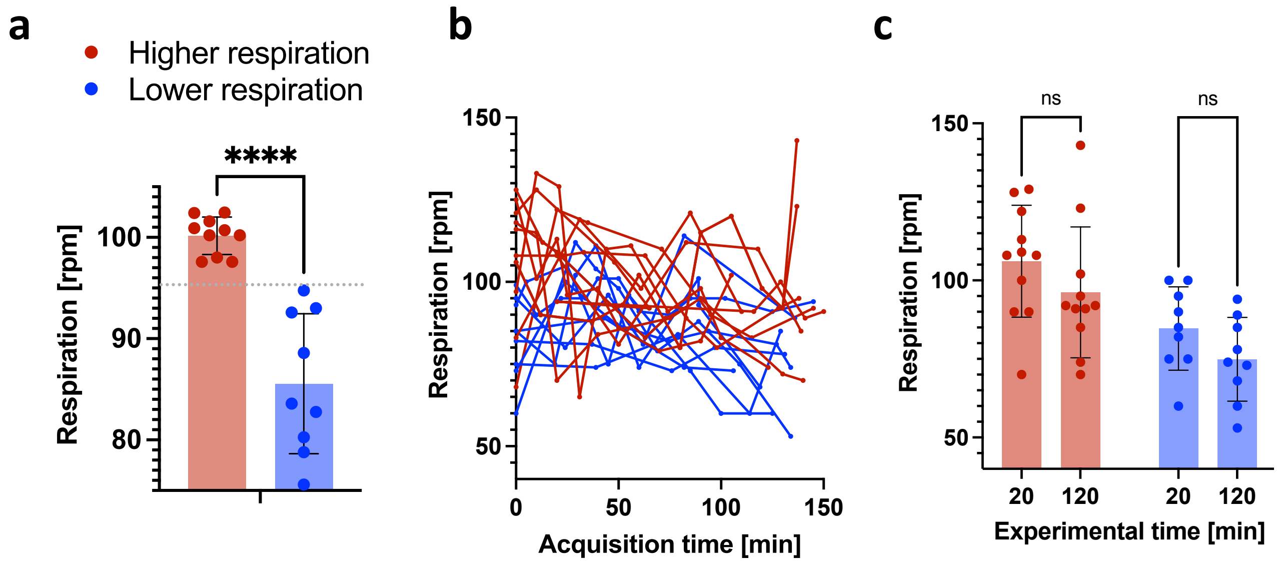

Supplementary Figure 4

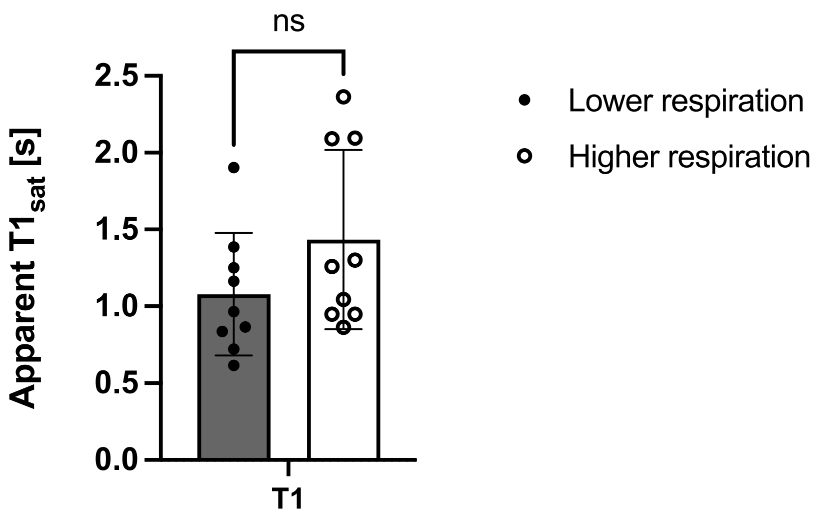

Supplement: Supplementary file 1 — Table S1. Absolute coefficient of variation in the test–retest analysis of individual 31P‐MRS metabolites. Calculated coefficients of variation (CVs) for each metabolite after 43 min of acquisition for “between‐group” and “between‐session” experiments and for 13‐min time points for “within‐session” experiments. Figure S1. Relationship of SNRPCr with increasing number of acquisitions. (a) Relationship between the SNR of phosphocreatine (SNRPCr) and number of acquisition averages (represented as acquisition time here). (b) Fitted parameters of a power equation (SNR = a·N b , where N is the number of acquisition) for individual mice. No sequence effect was observed for parameter b (F(2, 20) = 1.89, p = 0.18), but only for parameter a (F(2, 20) = 4.47, p = 0.02), RM one‐way ANOVA, *p < 0.05, Bonferroni’s post hoc test. Figure S2. Effect of long saturation time on saturation pulse bandwidth. Saturation pulse bandwidth comparison in phantom using BISTRO (green), HS2 pulse with fixed amplitude (orange) or sinc3 pulse with fixed amplitude (blue) at various saturation times. Figure S3. Stability of respiration during ST 31P‐MRS acquisition. (a) Breathing of higher and lower respiration groups. (b) Individual time courses of breathing rates for mice in the higher and lower respiration groups. (c) comparison between initial (first 20 min) and final (final 20 min) breathing rates for individual mice in each respiration groups. Group effect (F(1, 16) = 14, p = 0.002), time effect (F(1, 16) = 6.58, p = 0.02), repeated‐measure two‐way ANOVA, nonsignificant (ns) Bonferroni’s post hoc test. Figure S4. Apparent relaxation time T1 for the higher and lower respiration groups (ns, not significant). [file NBM-38-e70055-s001.pdf]
